# Safety and efficacy of ivabradine in the treatment of patients with acute myocardial infarction: a real-world propensity score matching study

**DOI:** 10.1093/ehjacc/zuag084

**Published:** 2026-06-15

**Authors:** Tianshu Gu, Wanlin Feng, Xian Shao, Jun-Yu Liu, Yu-Kun Zhang, Su-Tao Hu, Wen-Bo Dai, Tong Liu, Kang-Yin Chen

**Affiliations:** Tianjin Key Laboratory of Ionic-Molecular Function of Cardiovascular Disease, Department of Cardiology, Tianjin Institute of Cardiology, the Second Hospital of Tianjin Medical University, No. 23 Pingjiang Road, Hexi District, Tianjin 300211, China; Tianjin Key Laboratory of Ionic-Molecular Function of Cardiovascular Disease, Department of Cardiology, Tianjin Institute of Cardiology, the Second Hospital of Tianjin Medical University, No. 23 Pingjiang Road, Hexi District, Tianjin 300211, China; Division of Nephrology, National Clinical Research Center for Kidney Disease, State Key Laboratory of Organ Failure Research, Nanfang Hospital, Southern Medical University, Guangzhou, China; Tianjin Key Laboratory of Ionic-Molecular Function of Cardiovascular Disease, Department of Cardiology, Tianjin Institute of Cardiology, the Second Hospital of Tianjin Medical University, No. 23 Pingjiang Road, Hexi District, Tianjin 300211, China; Tianjin Key Laboratory of Ionic-Molecular Function of Cardiovascular Disease, Department of Cardiology, Tianjin Institute of Cardiology, the Second Hospital of Tianjin Medical University, No. 23 Pingjiang Road, Hexi District, Tianjin 300211, China; Tianjin Key Laboratory of Ionic-Molecular Function of Cardiovascular Disease, Department of Cardiology, Tianjin Institute of Cardiology, the Second Hospital of Tianjin Medical University, No. 23 Pingjiang Road, Hexi District, Tianjin 300211, China; Tianjin Key Laboratory of Ionic-Molecular Function of Cardiovascular Disease, Department of Cardiology, Tianjin Institute of Cardiology, the Second Hospital of Tianjin Medical University, No. 23 Pingjiang Road, Hexi District, Tianjin 300211, China; Tianjin Key Laboratory of Ionic-Molecular Function of Cardiovascular Disease, Department of Cardiology, Tianjin Institute of Cardiology, the Second Hospital of Tianjin Medical University, No. 23 Pingjiang Road, Hexi District, Tianjin 300211, China; Tianjin Key Laboratory of Ionic-Molecular Function of Cardiovascular Disease, Department of Cardiology, Tianjin Institute of Cardiology, the Second Hospital of Tianjin Medical University, No. 23 Pingjiang Road, Hexi District, Tianjin 300211, China

**Keywords:** Ivabradine, Acute myocardial infarction, Propensity score matching, Cardiac mortality, Real-world evidence

## Abstract

**Aims:**

Ivabradine, a selective If channel inhibitor, reduces heart rate without impairing myocardial contractility. Evidence regarding its safety and efficacy in the acute phase of acute myocardial infarction (AMI) remains limited and conflicting.

**Methods and results:**

We analysed data from 136 033 patients with AMI admitted to 82 hospitals in Tianjin, China, between 2010 and 2024. Primary outcomes were in-hospital mortality and 1-year cardiac mortality; secondary outcomes included 1-year all-cause mortality and other adverse events. Propensity score matching (PSM) was used to balance baseline characteristics between patients receiving ivabradine during hospitalization and controls. Logistic regression and Cox proportional hazards models were used to estimate outcome associations. Pre-specified subgroup analyses were conducted. Robustness was assessed using inverse probability of treatment weighting, overlap weighting, *E*-value analysis, complete-case analysis, treatment-pattern comparator analysis according to background beta-blocker use, and beta-blocker dose-intensity analysis. After PSM, in-hospital ivabradine use was associated with higher in-hospital mortality [aOR, 1.66 (95% CI, 1.19–2.32); *P* = 0.003], 1-year cardiac mortality [aHR, 1.22 (95% CI, 1.01–1.47); *P* = 0.043], and 1-year all-cause mortality [aHR, 1.19 (95% CI, 1.01–1.40); *P* = 0.040] in the fully adjusted model. No significant differences were observed in other secondary outcomes. Subgroup analyses suggested stronger associations among patients aged ≥65 years. Additional analyses showed that the mortality association was most pronounced among patients receiving ivabradine without concomitant beta-blocker. Sensitivity analyses yielded directionally consistent findings.

**Conclusion:**

In this large real-world AMI cohort, in-hospital ivabradine use was associated with higher in-hospital and 1-year mortality, without clear evidence of benefit for other secondary outcomes.

## Graphical Abstract

**Graphical Abstract:**
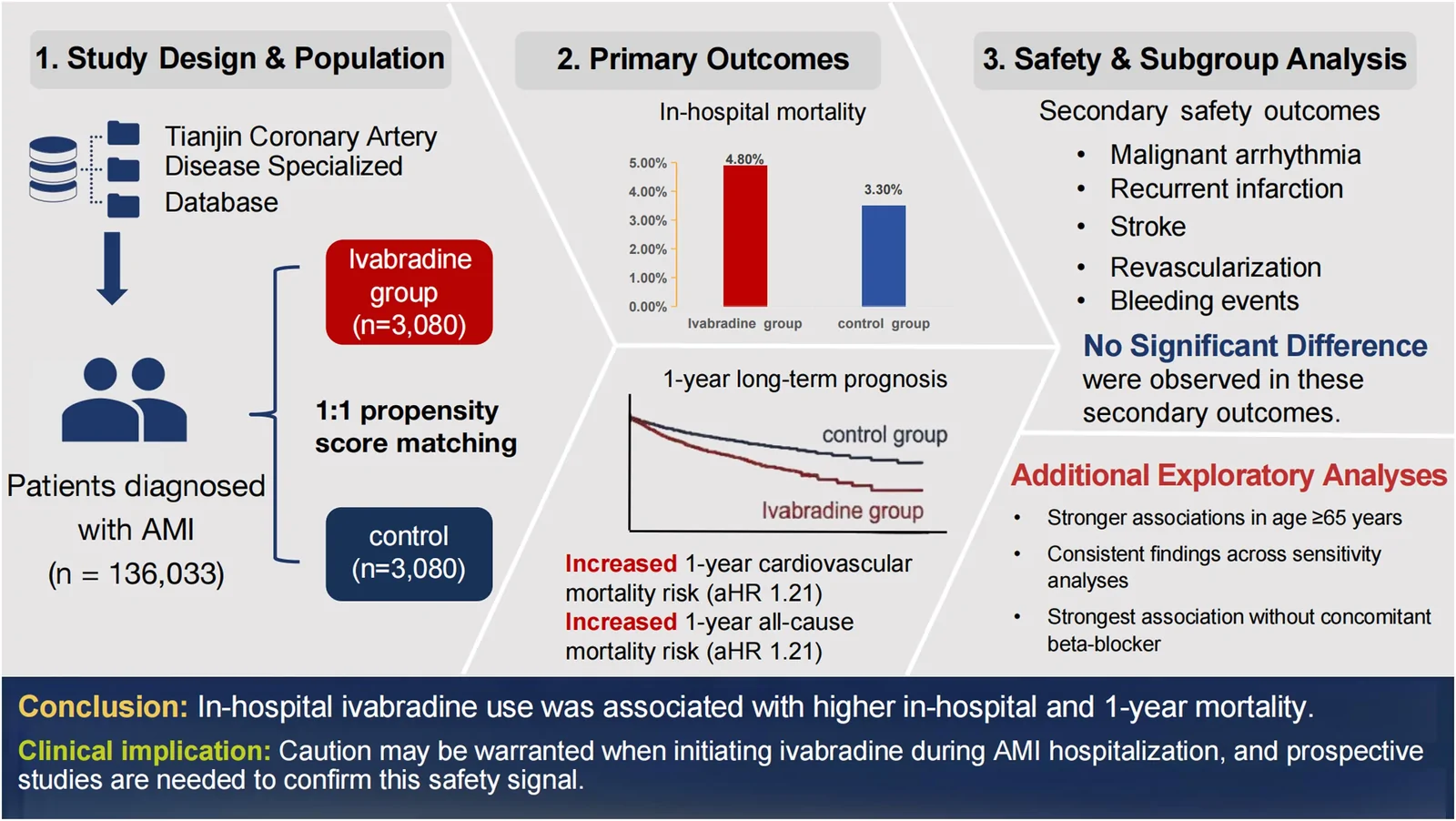
Study design, primary outcomes, and subgroup findings of ivabradine use in patients with acute myocardial infarction after 1:1 propensity score matching. In-hospital mortality was higher in the ivabradine group (4.8% vs. 3.3%), with higher risks of 1-year cardiac and all-cause mortality. No significant differences were observed in secondary outcomes, including malignant arrhythmias, recurrent myocardial infarction, stroke, revascularization, or BARC type 3 or higher bleeding. Additional exploratory analyses suggested that the mortality association was more pronounced among patients aged ≥65 years and strongest among patients receiving ivabradine without concomitant beta-blocker. aHR, adjusted hazard ratio; AMI, acute myocardial infarction; PSM, propensity score matching; TCADSD, Tianjin Coronary Artery Disease Specialized Database.

## Introduction

In the management of acute myocardial infarction (AMI), effective heart rate control is crucial for improving patient outcomes.^1^ The selection of beta-blockers for heart rate control in patients with myocardial infarction has been recommended by guidelines and is supported by substantial research evidence^2–4^. However, in cases where patients present with conditions such as hypotension that contraindicate the use of beta-blockers, alternative medications are required for achieving adequate heart rate control.^5^ Ivabradine, a novel selective heart rate-reducing agent, slows the heart rate by specifically inhibiting the If current in the sinoatrial node without affecting myocardial contractility^6–8^. Ivabradine has demonstrated a beneficial effect on prognosis in chronic heart failure and stable coronary artery disease (CAD).^9^ The 2021 ESC Guidelines for the diagnosis and treatment of acute and chronic heart failure recommend the use of ivabradine in symptomatic patients with heart failure with reduced ejection fraction who have a resting heart rate ≥70 beats per minute despite receiving guideline-directed medical therapy, to reduce the risk of heart failure hospitalization and cardiovascular mortality.^10^ However, evidence regarding the application of ivabradine in AMI patients, particularly its effectiveness and safety in real-world conditions, remains limited.

Previous studies on the use of ivabradine in AMI have predominantly consisted of small-scale prospective studies or randomized controlled trials (RCTs).^11,12^ While these studies have provided preliminary data on safety and efficacy, they are limited by factors such as single-centre design, small sample size, inconsistent treatment duration, varying follow-up period, and differing outcomes, which restrict the generalizability and applicability of their findings. Moreover, patient cohorts in randomized trials are often rigorously selected and may not accurately represent the clinical reality.

Consequently, the purpose of this study is to utilize a multicentre cohort of patients with AMI from the Tianjin Coronary Artery Disease Specialized Database (TCADSD, Tianjin, China), which includes data from 2010 to 2024. This study aims to assess the relationship between in-hospital use of ivabradine and short-term outcomes, such as in-hospital mortality, as well as 1-year outcomes, including cardiac mortality and other relevant outcomes.

## Methods

### Data source

The data for this study were sourced from the TCADSD. The details of the platform have been described in our previous publications.^13,14^ Briefly, the CAD specialized database includes patients who were hospitalized at least once between 1 January 2010, and 31 March 2024, with discharge diagnoses including CAD. All healthcare information for these patients is collected, including demographic characteristics, disease diagnoses, medication and non-medication prescriptions, examination and laboratory test results, surgical information, cost details, community medication and health examination information, and public health mortality information. The study protocol conforms to the ethical guidelines of the 1975 Declaration of Helsinki as reflected in *a priori* approval by the Clinical Research Ethics Committee of the Second Hospital of Tianjin Medical University (KY2023052-01), which waived the requirement for informed patient consent. The data that support the findings of this study are available from the corresponding author upon reasonable request. The reporting of this study followed the Strengthening the Reporting of Observational Studies in Epidemiology (STROBE) statement, and the completed STROBE checklist is provided in the Supplementary Material.^15^

### Study design and patient population

The identification of patients with AMI was based on discharge diagnoses from the TCADSD. Within the TCADSD, diagnoses and procedures are coded according to the International Classification of Diseases, Tenth Revision, Clinical Modification (ICD-10-CM), as well as the Procedure Coding System (ICD-10-PCS). We extracted patients with a primary discharge diagnosis of AMI from the database to form the primary AMI database, using ICD-10-CM codes I21 and I22. *Figure 1* presents the study design flowchart. Patients older than 100 years and younger than 18 years were excluded from the study. Additionally, individuals diagnosed with bradycardia, sinus arrest, atrioventricular block, sinoatrial node dysfunction, or atrial fibrillation were also excluded due to contraindications and potential adverse reactions to ivabradine. Because the objective of the present study was to evaluate ivabradine use during the acute hospitalization for AMI, patients who initiated ivabradine only after discharge were excluded to preserve a clear in-hospital exposure definition and to avoid exposure misclassification or time-related bias. Patients with missing data for the essential variables of sex or age were also excluded from the analysis.

**Figure 1:**
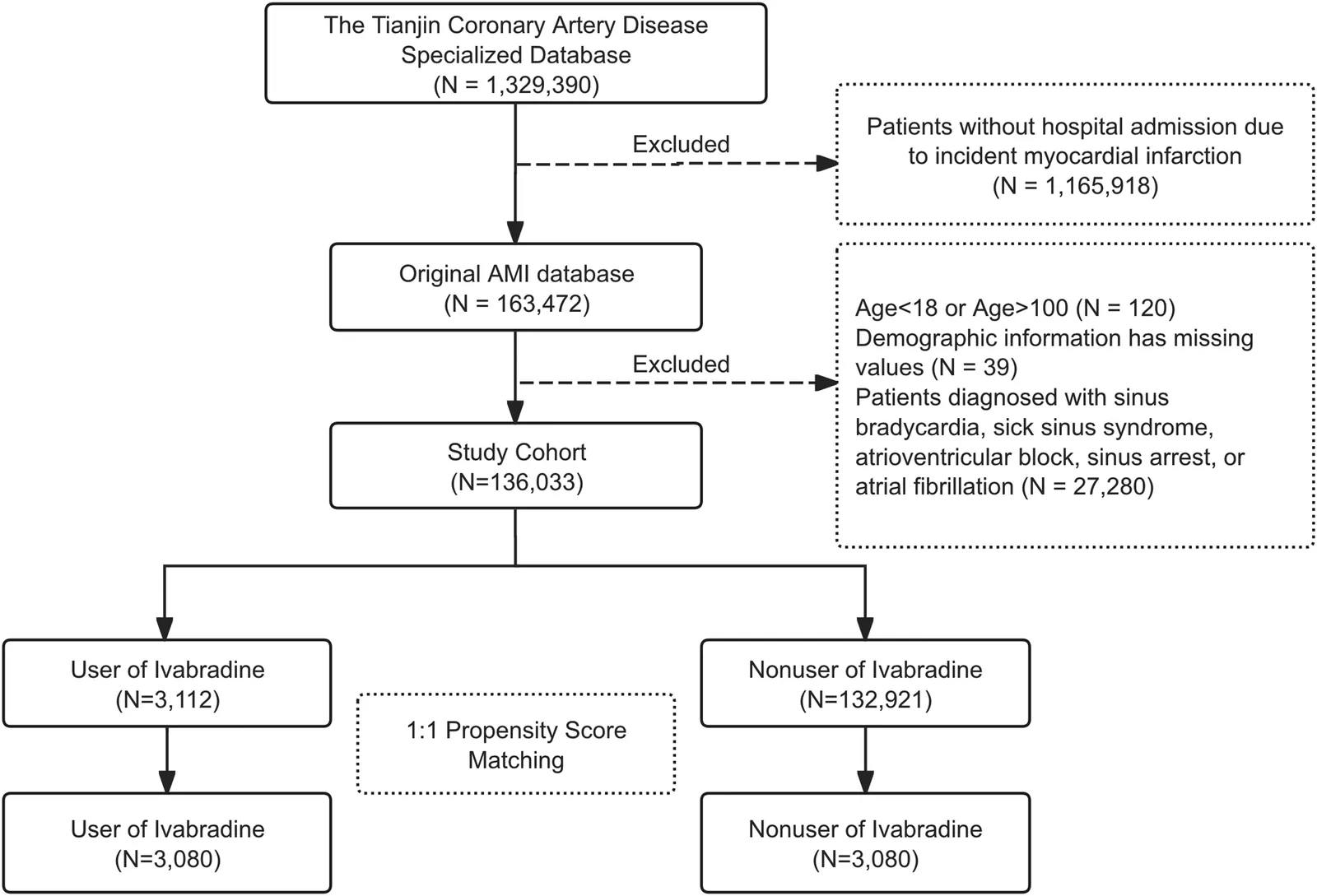
The study design flowchart.

### Outcome assessments

The study outcomes encompassed both in-hospital adverse events and 1-year adverse outcomes. The primary outcome measures included in-hospital mortality and 1-year cardiac mortality. Secondary in-hospital adverse outcomes included malignant arrhythmias that were not included in the exclusion criteria for the study cohort, specifically ventricular tachycardia, ventricular fibrillation, and ventricular flutter. Secondary 1-year outcomes included all-cause mortality, follow-up myocardial infarction, revascularization events, stroke events and Bleeding Academic Research Consortium (BARC) type 3 or higher bleeding events.

Follow-up myocardial infarctions were defined as subsequent hospital admissions for AMI recorded in the TCADSD after the baseline event. Stroke was defined as new hospital admissions within 1 year due to either new cerebral infarction or haemorrhage. Revascularization events were defined as any revascularization procedure performed on any segment of the coronary artery, including branch vessels, after discharge from the baseline AMI. BARC type 3 or higher bleeding events were defined as bleeding events that meet the BARC classification of types 3–5, as recorded in the TCADSD.

### Statistical analysis

Continuous variables were presented as mean ± standard deviation (SD), while categorical variables were expressed as absolute numbers and proportions. The Wilcoxon test was used to compare continuous variables between groups, and the chi-square test was used for categorical variables. Missing values for the additional baseline variables were handled using multiple imputation by chained equations. Multiple imputed datasets were generated and estimates were combined using Rubin’s rules. The multiply-imputed dataset was used as the primary analytic dataset; complete-case analysis was performed as a sensitivity analysis to confirm that the findings were not driven by the imputation approach. We employed propensity score matching (PSM) to address baseline imbalances and variables that could affect treatment allocation. Details of the pre- and post-matching standardized mean differences (SMDs) are shown in Supplementary material online, *Figure S1*. A greedy nearest neighbour matching method was used to match patients exposed to ivabradine during hospitalization with those unexposed at a 1:1 ratio. The calliper width was set to 0.02 of the logit of the SD of the propensity score. Baseline balance was assessed using absolute SMD, with an absolute SMD ≤0.1 indicating adequate balance between the cohorts.

We applied multivariable regression models to evaluate the association between in-hospital ivabradine use and adverse clinical outcomes. For in-hospital adverse events, logistic regression models were used, while Cox proportional hazards models were employed for 1-year outcomes. In the unmatched cohort, models were adjusted for the following covariates: sex, STEMI status, age, Killip class, peripheral vascular disease, cerebrovascular disease, hypertension, diabetes mellitus, in-hospital beta-blocker, angiotensin-converting enzyme inhibitors/angiotensin receptor blockers (ACEI/ARBs), calcium-channel blocker, statin, and diuretic use. In the PS-matched cohort, fully adjusted models were fitted to estimate odds ratios (aORs) or hazard ratios (aHRs) with corresponding 95% confidence intervals (CIs). Four multivariable models were specified for the PS-matched cohort to evaluate the robustness of the association: Model 1 included sex, age, STEMI status, Killip class, the Charlson Comorbidity Index, and the Hospital Frailty Risk Score; Model 2 added the major comorbidities at admission; Model 3 added in-hospital pharmacotherapy; and Model 4 additionally adjusted for the location of the index AMI and additional baseline biomarkers. Kaplan–Meier curves were generated to visualize survival outcomes, and significance was evaluated using the log-rank test. Pre-specified subgroup analyses were conducted to explore potential effect modification by age (<65 vs. ≥ 65 years), sex, AMI subtype (STEMI vs. NSTEMI), Killip classification, hypertension, diabetes mellitus, and in-hospital β-blocker use.

In addition to the pre-specified analyses described above, we performed several supplementary analyses to support and contextualize the primary findings. First, sensitivity analyses were performed using inverse probability of treatment weighting (IPTW) targeting the average treatment effect (ATE) and overlap weighting targeting the ATE in the overlap population (ATO). For each approach, we fitted doubly robust outcome models that combined the weights with multivariable adjustment for pre-specified confounders. Time-to-event outcomes were analysed using survey-weighted Cox models; in-hospital mortality was analysed using survey-weighted logistic regression with a quasibinomial family to obtain odds ratios. To assess the potential impact of unmeasured confounding on the primary PSM fully adjusted (Model 4) estimates, we computed *E*-values for both the point estimates and the lower limits of their 95% CIs. Second, a treatment-pattern comparator analysis was performed using patients receiving a beta-blocker without ivabradine as the reference group, comparing them with patients receiving ivabradine without a concomitant beta-blocker and with patients receiving ivabradine on top of a beta-blocker. Third, to demonstrate the prognostic validity of the dataset by examining established AMI risk factors, we performed univariable and multivariable logistic and Cox regression analyses in the overall original cohort that simultaneously included age, sex, STEMI status, Killip class, the Charlson Comorbidity Index, the Hospital Frailty Risk Score, comorbidities, in-hospital PCI, and guideline-directed pharmacotherapy. Fourth, among beta-blocker users with available dose data, we evaluated beta-blocker dose intensity according to achievement of ≥50% or ≥100% of guideline- or trial-based target doses and further assessed its association with in-hospital and 1-year mortality.

Two-sided *P* values of < 0.05 were considered statistically significant. The statistical tests were performed using R programming version 4.4.1 (The R Foundation for Statistical Computing, Vienna, Austria).

## Results

### Population characteristics

A total of 136 033 patients [age: 64.67 (±12.65) years, female: 42 819 (32.2%)] with AMI met the inclusion criteria for the study, of whom 3112 (2.29%) received ivabradine during their hospital stay. In the overall cohort (*Table 1*), the distribution of sex and age was balanced between patients with AMI who did and did not receive ivabradine during hospitalization. Patients in the ivabradine group had a lower proportion of STEMI and a higher Killip class. The prevalence of cardiovascular disease was higher in the ivabradine group, with elevated rates of comorbidities such as peripheral vascular disease, cerebrovascular disease, and diabetes. There was no significant difference in the prevalence of hypertension between the two groups. In terms of in-hospital treatment, the proportion of patients receiving primary percutaneous coronary intervention (PCI) was lower in the ivabradine group than control group (38.7% vs. 41.2%), which we hypothesize is related to the lower proportion of STEMI cases. However, the final in-hospital PCI rate did not differ substantially between the two groups. Regarding in-hospital medication usage, the administration of antiplatelet agents, CCBs, beta-blockers, ACEI/ARBs, diuretics, and statins was notably higher in the ivabradine group. After 1:1 PSM, 3080 ivabradine-treated patients were matched to 3080 controls, with all included covariates achieving adequate balance (SMD <0.10).

**Table 1:** Baseline characteristics of patients with acute myocardial infarction according to in-hospital ivabradine use before and after propensity score matching

	Unmatched cohort		SMD	PS-matched cohort		SMD
Characters	No Ivabradine (*n* = 132 921)	Ivabradine (*n* = 3112)		No Ivabradine (*n* = 3080)	Ivabradine (*n* = 3080)	
Female, *n* (%)	42 819 (32.2)	1002 (32.2)	<0.001	996 (32.3)	994 (32.3)	0.001
Age (mean (SD))	64.67 (12.65)	64.78 (13.30)	0.008	64.70 (12.57)	64.79 (13.32)	0.007
STEMI, *n* (%)	67 526 (50.8)	1069 (34.4)	0.337	1079 (35.0)	1064 (34.5)	0.010
Anterior MI	33 591 (25.3)	1093 (35.1)	0.286	1050 (34.1)	1080 (35.1)	0.054
Inferior MI	29 039 (21.8)	398 (12.8)		445 (14.4)	393 (12.8)	
Other walls	1828 (1.4)	34 (1.1)		27 (0.9)	34 (1.1)	
Not specified	68 463 (51.5)	1587 (51.0)		1558 (50.6)	1573 (51.1)	
CCI [mean (SD)]	1.22 (1.94)	2.09 (2.39)	0.401	1.96 (2.42)	2.07 (2.36)	0.046
HFRS [mean (SD)]	4.91 (2.99)	5.93 (3.45)	0.316	5.67 (3.55)	5.91 (3.45)	0.069
KILLIP, *n* (%)			0.460			0.015
I	88 491 (66.6)	1405 (45.1)		1378 (44.7)	1398 (45.4)	
II	25 900 (19.5)	836 (26.9)		849 (27.6)	831 (27.0)	
III	13 296 (10.0)	657 (21.1)		645 (20.9)	644 (20.9)	
IV	5234 (3.9)	214 (6.9)		208 (6.8)	207 (6.7)	
*Previous cardiovascular disease, n (%)*						
Ischemic heart disease	63 152 (47.5)	1960 (63.0)	0.315	1862 (60.5)	1933 (62.8)	0.047
Angina	22 603 (17.0)	727 (23.4)	0.159	781 (25.4)	711 (23.1)	0.053
Myocardial Infarction	12 470 (9.4)	407 (13.1)	0.117	371 (12.0)	397 (12.9)	0.026
*Comorbidities, n (%)*						
Peripheral vascular disease	11 116 (8.4)	577(18.5)	0.302	559 (18.1)	564 (18.3)	0.004
Cerebrovascular disease	30 633 (23.0)	877 (28.2)	0.118	871 (28.3)	861 (28.0)	0.007
Gastrointestinal bleeding	3989 (3.0)	97 (3.1)	0.007	108 (3.5)	93 (3.0)	0.027
Diabetes mellitus	44 536 (33.5)	1422 (45.7)	0.251	1295 (42.0)	1404 (45.6)	0.071
Hypertension	83 694 (63.0)	1900 (61.1)	0.039	1921 (62.4)	1881 (61.1)	0.027
*Laboratory tests [mean (SD)]*						
eGFR	80.23 (31.69)	76.49 (34.57)	0.113	75.94 (32.96)	76.60 (34.51)	0.019
Haemoglobin	137.83 (26.41)	133.95 (27.04)	0.145	136.14 (26.39)	134.06 (27.05)	0.078
CK-MB	107.33 (185.50)	118.72 (191.55)	0.060	128.50 (201.52)	119.18 (192.26)	0.047
*Inpatient medications, n (%)*						
Primary PCI	54 788 (41.2)	1205 (38.7)	0.051	1249 (40.6)	1201 (39.0)	0.032
In-hospital PCI	73 905 (55.6)	1784 (57.3)	0.035	1803 (58.5)	1769 (57.4)	0.022
Aspirin	116 262 (87.5)	2825 (90.8)	0.106	2792 (90.6)	2798 (90.8)	0.007
P2Y12 inhibitors	119 541 (89.9)	3035 (97.5)	0.317	2949 (95.7)	3003 (97.5)	0.097
CCBs	25 444 (19.1)	600 (19.3)	0.004	576 (18.7)	591 (19.2)	0.012
Beta-blockers	90 830 (68.3)	2510 (80.7)	0.286	2489 (80.8)	2481 (80.6)	0.007
ACEI/ARBs	83 692 (63.0)	2204 (70.8)	0.168	2158 (70.1)	2173 (70.6)	0.011
Statins	119 648 (90.0)	2994 (96.2)	0.246	2973 (96.5)	2962 (96.2)	0.019
Diuretics	64 905 (48.8)	2390 (76.8)	0.605	2361 (76.7)	2358 (76.6)	0.002
NOACs	976 (0.7)	89 (2.6)	0.144	75 (2.4)	77 (2.5)	0.004

Data are presented as mean ± SD or *n* (%). Standardized mean differences were used to assess between-group balance before and after propensity score matching, with an absolute standardized mean difference <0.10 indicating adequate balance. The ivabradine group included patients who received ivabradine during the index hospitalization for acute myocardial infarction; the no-ivabradine group included patients who did not receive ivabradine during the index hospitalization. ACEI, angiotensin-converting enzyme inhibitor; AMI, acute myocardial infarction; ARB, angiotensin receptor blocker; CCB, calcium-channel blocker; CCI, Charlson Comorbidity Index; CK-MB, creatine kinase-MB; eGFR, estimated glomerular filtration rate; HFRS, Hospital Frailty Risk Score; NOAC, non-vitamin K antagonist oral anticoagulant; PCI, percutaneous coronary intervention; PSM, propensity score matching; SD, standard deviation; SMD, standardized mean difference; STEMI, ST-segment elevation myocardial infarction.

### Clinical outcomes

*Table 2* illustrates the rates of in-hospital and 1-year adverse events for the cohort, both prior to and following PSM. In both pre- and post-matching cohorts, the ivabradine group exhibited significantly higher risks of in-hospital mortality [unmatched cohort: 4.8% vs. 3.6%, aOR, 1.67 (95% CI, 1.36–2.04); *P* < 0.001; PS-matched cohort: 4.8% vs. 3.3%, OR, 1.50 (95% CI, 1.16–1.94); *P* = 0.002], 1-year all-cause mortality [unmatched cohort: 13.4% vs. 9.7%, aHR, 1.26 (95% CI, 1.13–1.40); *P* < 0.001; PS-matched cohort: 13.3% vs. 11.1%, HR, 1.20 (95% CI, 1.04–1.38); *P* = 0.014], and 1-year cardiac mortality [unmatched cohort: 10.1% vs. 7.5%, aHR, 1.25 (95% CI, 1.10–1.41); *P* < 0.001; PS-matched cohort: 10.1% vs. 8.4%, HR, 1.20 (95% CI, 1.02–1.42); *P* = 0.031] compared with the control group. *Figure 2* presents the Kaplan–Meier curves for the cumulative incidence of 1-year all-cause mortality and 1-year cardiac mortality.

**Figure 2:**
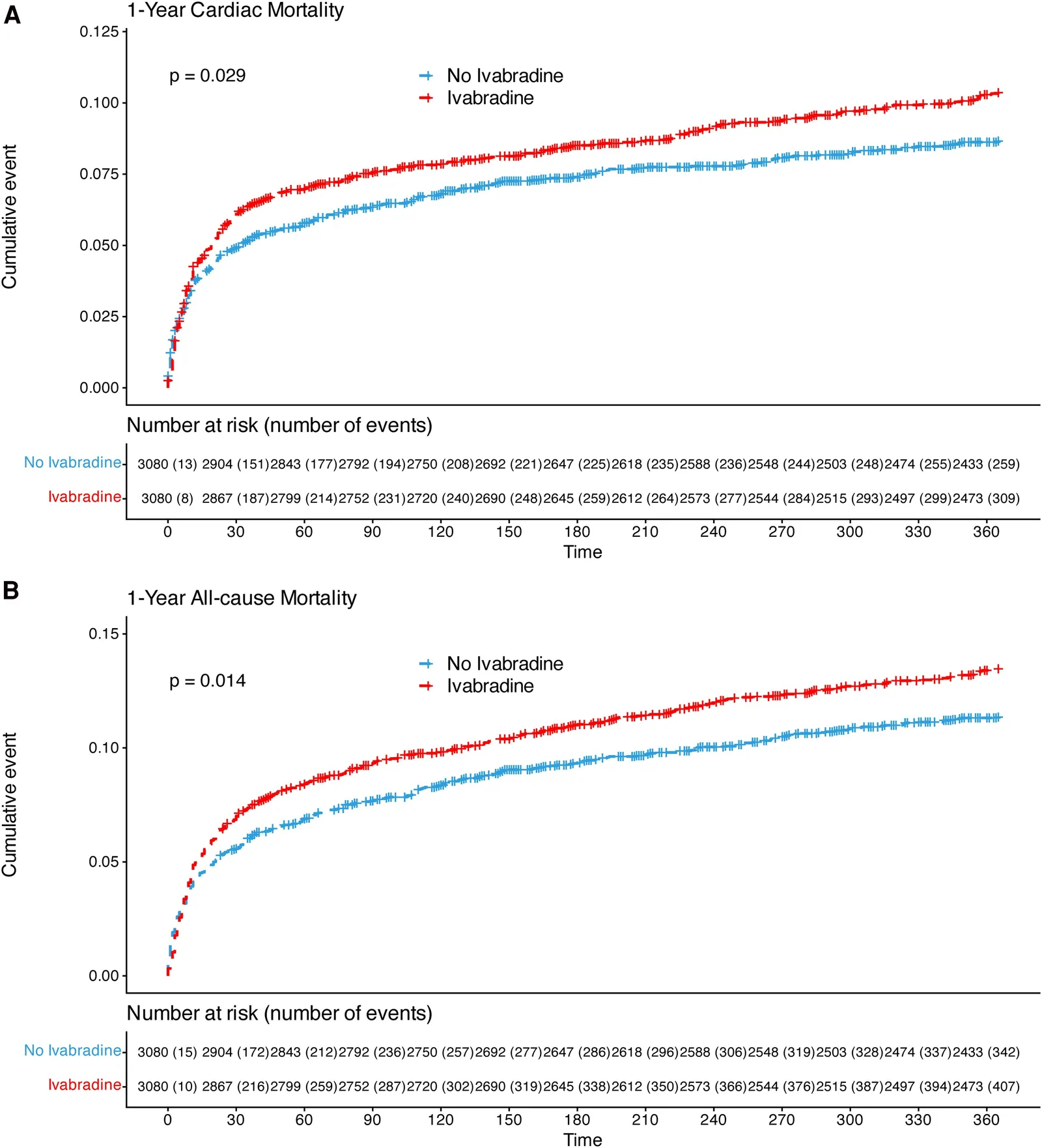
Kaplan–Meier curves for 1-year cardiac and all-cause mortality after propensity score matching. Kaplan–Meier curves for 1-year cardiac mortality (*A*) and 1-year all-cause mortality (*B*) in the propensity-score-matched cohort. The ivabradine group included patients who received ivabradine during the index hospitalization for acute myocardial infarction; the no-ivabradine group included matched patients who did not receive ivabradine during the index hospitalization. *P* values were calculated using the log-rank test. AMI, acute myocardial infarction.

**Table 2:** In-hospital and 1-year clinical outcomes according to in-hospital ivabradine use before and after propensity score matching

Outcomes	Unmatched cohort		PS-matched cohort	
In-hospital adverse events	Event rate (%)	aOR (95% CI), *P*	Event rate (%)	OR (95% CI), *P*
In-hospital mortality	4.8 vs. 3.6	1.67 (1.36–2.04), *P* < 0.001	4.8 vs. 3.3	1.50 (1.16–1.94), *P* = 0.002
Rapid malignant arrhythmias	5.7 vs. 3.7	1.14 (0.95–1.36), *P* = 0.147	5.7 vs. 4.9	1.16(0.93–1.45), *P* = 0.200

1-Year adverse event	Event rate (%)	aHR (95% CI), *P*	Event rate (%)	HR (95% CI), *P*
Cardiac mortality	10.1 vs. 7.5	1.25 (1.10–1.41), *P* < 0.001	10.1 vs. 8.4	1.20 (1.02–1.42), *P* = 0.031
All-cause mortality	13.4 vs. 9.7	1.26 (1.13–1.40), *P* < 0.001	13.3 vs. 11.1	1.20 (1.04–1.38), *P* = 0.014
Myocardial infarction	5.5 vs. 4.1	1.03 (0.86–1.22), *P* = 0.771	5.3 vs. 5.6	0.96 (0.78–1.19), *P* = 0.703
Stroke	2.9 vs. 2.5	1.25 (0.98–1.58), *P* = 0.066	2.9 vs. 2.6	1.13 (0.83–1.53), *P* = 0.451
Revascularization	15.6 vs. 16.0	1.09 (0.98–1.20), *P* = 0.089	15.7 vs. 16.0	0.95 (0.87–1.12), *P* = 0.825
BARC ≥ 3 bleeding	0.9 vs. 0.6	1.25 (0.86–1.82), *P* = 0.245	0.9 vs. 0.5	1.87 (0.99– 3.51), *P* = 0.052

Event rates are presented as ivabradine vs. no ivabradine. Logistic regression was used for in-hospital outcomes, and Cox proportional hazards models were used for 1-year outcomes. In the unmatched cohort, estimates were adjusted for pre-specified baseline covariates and are reported as adjusted odds ratios or adjusted hazard ratios. In the propensity-score-matched cohort, estimates were derived from univariable models within the matched sample and are reported as odds ratios or hazard ratios. All estimates are expressed for in-hospital ivabradine use vs. no in-hospital ivabradine use. aHR, adjusted hazard ratio; aOR, adjusted odds ratio; BARC, Bleeding Academic Research Consortium; CI, confidence interval; HR, hazard ratio; MI, myocardial infarction; OR, odds ratio; PSM, propensity score matching.

For other secondary outcomes, there were no statistically significant differences between the two groups after PSM, including rapid malignant arrhythmias (unmatched cohort: 5.7% vs. 3.7%, aOR, 1.14; *P* = 0.147; PS-matched cohort: 5.7% vs. 4.9%, OR, 1.16; *P* = 0.200), 1-year myocardial infarction (unmatched cohort: 5.5% vs. 4.1%, aHR, 1.03; *P* = 0.771; PS-matched cohort: 5.3% vs. 5.6%, HR, 0.96; *P* = 0.703), stroke (unmatched cohort: 2.9% vs. 2.5%, aHR, 1.25; *P* = 0.066; PS-matched cohort: 2.9% vs. 2.6%, HR, 1.13; *P* = 0.451), revascularization (unmatched cohort: 15.6% vs. 16.0%, aHR, 1.09; *P* = 0.089; PS-matched cohort: 15.7% vs. 16.0%, HR, 0.95; *P* = 0.825), and BARC ≥ 3 bleeding (unmatched cohort: 0.9% vs. 0.6%, aHR, 1.25; *P* = 0.245; PS-matched cohort: 0.9% vs. 0.5%, HR, 1.87; *P* = 0.052).

After performing regression analysis adjusted for potential confounding factors, the results demonstrated a consistent trend across the four models, which aligned with the observed event rates. In the post-PSM cohort, the use of ivabradine continued to demonstrate a significantly higher risk of in-hospital mortality, 1-year cardiac mortality, and 1-year all-cause mortality, with the same trend observed in all additional regression models (*Table 3*). Notably, the use of ivabradine in-hospital did not significantly impact the occurrence of other secondary outcomes (*Figure 3*).

**Figure 3:**
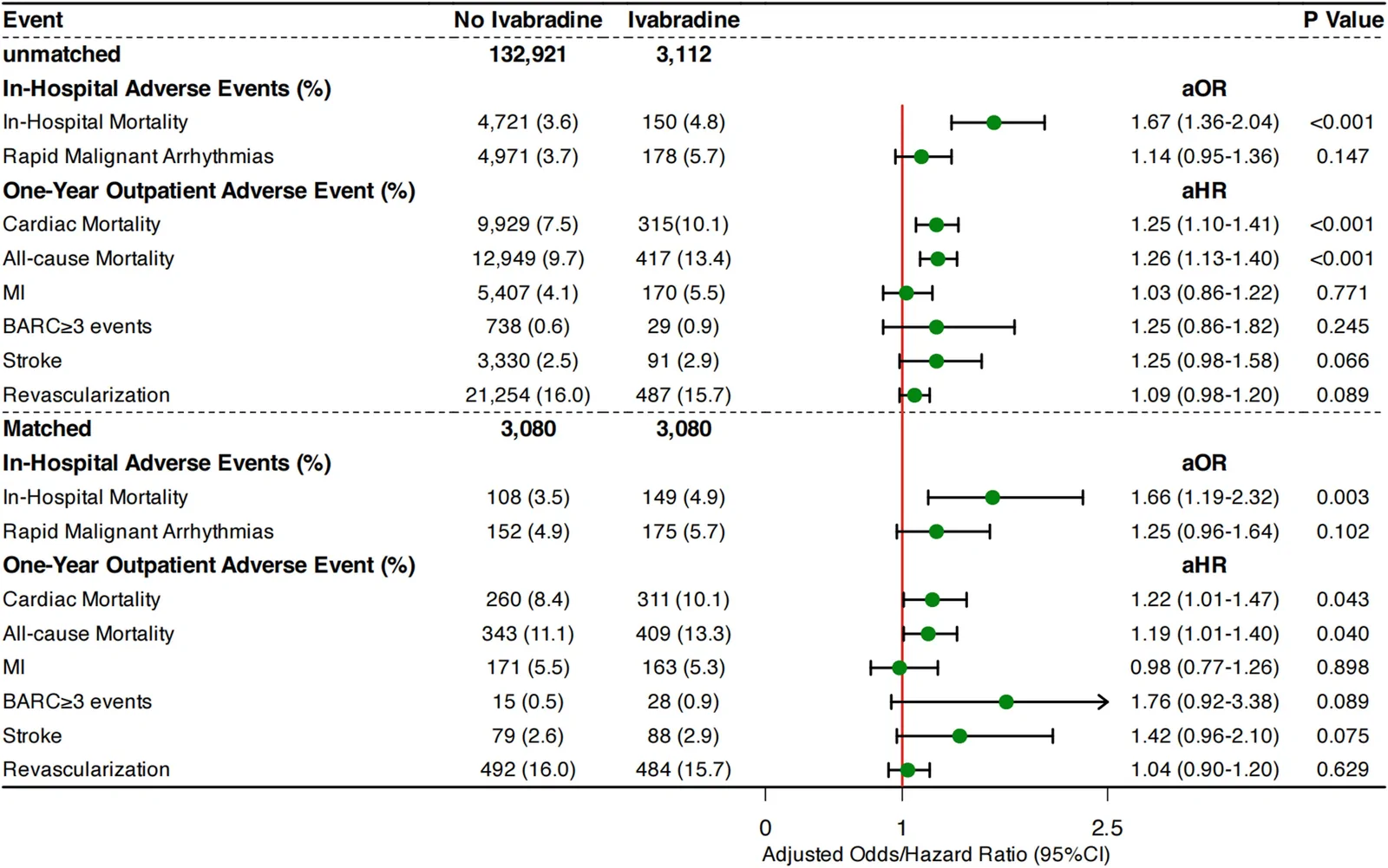
Fully adjusted associations between in-hospital ivabradine use and clinical outcomes before and after propensity score matching. Forest plot showing the fully adjusted associations between in-hospital ivabradine use and in-hospital and 1-year clinical outcomes in the unmatched and propensity-score-matched cohorts. Logistic regression was used for in-hospital outcomes, and Cox proportional hazards models were used for 1-year outcomes. Estimates are expressed for in-hospital ivabradine use vs. no in-hospital ivabradine use. Fully adjusted models included demographics, STEMI status, Killip class, the Charlson Comorbidity Index, Hospital Frailty Risk Score, comorbidities, in-hospital pharmacotherapy, infarct location, and baseline biomarkers. Squares indicate point estimates, and horizontal lines indicate 95% confidence intervals. aHR, adjusted hazard ratio; aOR, adjusted odds ratio; BARC, Bleeding Academic Research Consortium; CI, confidence interval; MI, myocardial infarction; PSM, propensity score matching; STEMI, ST-segment elevation myocardial infarction.

**Table 3:** Multivariable regression models for the association between in-hospital ivabradine use and mortality outcomes in the propensity-score-matched cohort

	In-hospital mortality		1-Year All-cause mortality		1-Year cardiac mortality	
	aOR (95% CI)	*P* value	aHR (95%CI)	*P* value	aHR (95%CI)	*P* value
Model 1	1.52 (1.16–2.01)	0.003	1.19 (1.02–1.37)	0.023	1.18 (1.00–1.40)	0.044
Model 2	1.51 (1.15–2.00)	0.003	1.18 (1.02–1.37)	0.029	1.18 (1.00–1.39)	0.050
Model 3	1.65 (1.24–2.20)	<0.001	1.21 (1.03–1.40)	0.012	1.21 (1.01–1.44)	0.034
Model 4	1.66 (1.19–2.32)	0.003	1.19 (1.01–1.40)	0.040	1.22 (1.01–1.47)	0.043

Logistic regression models were used to analyse in-hospital adverse events; Cox proportional hazards models were utilized for assessing 1-year adverse outcomes. Estimates are expressed for in-hospital ivabradine use vs. no in-hospital ivabradine use. Model 1 adjusted for sex, age, STEMI status, Killip class, the Charlson Comorbidity Index, and the Hospital Frailty Risk Score. Model 2 additionally adjusted for comorbidities at admission. Model 3 additionally adjusted for in-hospital pharmacotherapy. Model 4 additionally adjusted for infarct location and baseline biomarkers, including creatine kinase-MB, estimated glomerular filtration rate, and haemoglobin. aOR indicates adjusted odds ratio; aHR, adjusted hazard ratio; CI, confidence interval; STEMI, ST-segment elevation myocardial infarction.

### Subgroup analysis

The results of these subgroup analyses remained consistent across the three events (see Supplementary material online, *Figure S2*). Significant interactions were observed for age across all three outcomes, with stronger associations in patients aged ≥65 years than in those aged <65 years: in-hospital mortality (*P* for interaction = 0.032), 1-year cardiac mortality (*P* for interaction = 0.022), and 1-year all-cause mortality (*P* for interaction = 0.014). Additional interactions were observed for sex in 1-year cardiac mortality (*P* for interaction = 0.043) and for beta-blocker use in 1-year all-cause mortality (*P* for interaction = 0.033). No significant interactions were observed for AMI subtype, Killip class, diabetes, or hypertension.

### Sensitivity analyses

Two complementary causal-inference specifications applied to the overall pre-matching cohort—IPTW and overlap weighting—yielded effect estimates that were directionally consistent and similar in magnitude to the primary PSM Model 4 results across all three primary outcomes (see Supplementary material online, *Table S1*), indicating that the observed harm signals were not specific to a single estimand. *E*-values for the primary PSM Model 4 estimates across the three primary outcomes are presented in Supplementary material online, *Table S2*.

In the treatment-pattern comparator analysis using beta-blocker–only patients as the reference, the harm signal was most pronounced among patients receiving ivabradine monotherapy without a concomitant beta-blocker (in-hospital aOR 3.20, 95% CI 2.38–4.24; 1-year cardiac mortality aHR 1.87, 95% CI 1.55–2.25; 1-year all-cause mortality aHR 2.07, 95% CI 1.77–2.42; Supplementary material online, *Table S3*).

Multivariable analyses of established AMI prognostic variables showed clinically plausible associations with mortality outcomes. In-hospital PCI was strongly associated with lower in-hospital mortality (aOR, 0.38), 1-year cardiac mortality (aHR, 0.31), and 1-year all-cause mortality (aHR, 0.31). Higher Killip class, age, comorbidity burden, diabetes mellitus, and cerebrovascular disease were generally associated with worse outcomes, whereas beta-blocker, ACEI/ARB, and statin use showed protective associations in the expected direction (see Supplementary material online, *Tables S4*–*SS6*).

Among beta-blocker users, target-dose achievement was uniformly low: only 30.1% of non-ivabradine users and 33.0% of ivabradine users reached ≥50% of the guideline-recommended dose, and only 3.1% and 4.0%, respectively, reached ≥100% of the target dose (see Supplementary material online, *Table S7*). The prognostic impact of non-optimal beta-blocker dosing was modest and similar between dose strata (see Supplementary material online, *Table S8*).

The complete-case sensitivity analysis included 82 484 patients in the original cohort, including 2277 patients receiving in-hospital ivabradine. After 1:1 PSM, 2254 ivabradine-treated patients were matched to 2254 controls. The results were directionally and quantitatively concordant with those from the primary multiple-imputation analysis (see Supplementary material online, *Tables S9* and *S10*).

## Discussion

In this large, multicentre, real-world cohort of patients hospitalized with AMI, we observed several key findings. First, in-hospital ivabradine use was associated with higher risks of in-hospital mortality, 1-year all-cause mortality, and 1-year cardiac mortality. These associations were consistent across unmatched and propensity score-matched analyses and were further supported by multiple sensitivity analyses. Second, ivabradine use was not associated with a significant increase in the risk of other major adverse cardiovascular or bleeding events, including malignant arrhythmias, myocardial infarction, stroke, revascularization, and major bleeding.

Our findings provide the first large-scale, real-world evidence regarding the safety profile of ivabradine in patients with AMI. Previous randomized trials investigating ivabradine in the acute setting were limited by small sample sizes, heterogeneous populations, and short follow-up durations. Some suggested potential benefits in heart rate control, but clinical endpoints were rarely assessed and often inconclusive.^11,12^ In contrast to these prior small studies, the present study identified an association between in-hospital ivabradine use and higher in-hospital and 1-year mortality in a broad real-world AMI population. The disparity between these RCTs and this real-world study underscores the importance of large-scale observational studies to capture the complexities inherent in clinical practice. Moreover, our findings are broadly consistent with prior evidence suggesting that ivabradine does not necessarily translate heart rate reduction into improved clinical outcomes across all cardiovascular settings. A comparative study among patients with atrial fibrillation reported that those administered ivabradine were at an elevated risk of hospitalization due to heart failure, as well as a higher risk of all-cause mortality.^16^ The SIGNIFY study indicated that ivabradine did not mitigate the risk of major adverse cardiovascular events in stable CAD patients without clinical heart failure, and it was associated with an increased incidence of bradycardia across all angina subgroups classified by the Canadian Cardiovascular Society.^17^ Furthermore, a study based on the SIGNIFY population found that the treatment of stable CAD patients with ivabradine was associated with a higher incidence of atrial fibrillation, without improving the prognosis in patients without clinical heart failure.^18^

The mechanisms underlying the observed association between ivabradine use and poorer outcomes in AMI remain incompletely understood. One study in patients with stable CAD showed that 6-month treatment with ivabradine reduced heart rate but was associated with increased stroke volume and central systolic pressure.^19^ This increase in central blood pressure may attenuate the potential benefits of heart rate reduction. Elevated central aortic blood pressure can increase left ventricular afterload and has been associated with adverse cardiovascular outcomes. In the Conduit Artery Function Evaluation (CAFE) study, each 10 mmHg increase in central aortic pulse pressure was associated with a 23% increase in the combined risk of cardiovascular events, procedures, or renal impairment.^20^ Therefore, altered central haemodynamics may represent one possible explanation for the observed association in AMI patients, although this mechanism remains speculative because the available haemodynamic evidence is mainly derived from stable CAD rather than acute AMI. Although small haemodynamic studies have suggested potential benefits of ivabradine in selected patients with cardiogenic shock, these studies were not designed to assess mortality.^21^ Moreover, a meta-analysis of randomized trials in critically ill patients, including AMI and cardiogenic shock populations, did not demonstrate a clear mortality benefit of in-hospital ivabradine use.^22^ Therefore, whether ivabradine has favourable or unfavourable haemodynamic effects in the acute AMI setting remains uncertain and requires dedicated prospective evaluation.

Beyond potential haemodynamic mechanisms, our subgroup analyses suggested exploratory heterogeneity in the association between ivabradine use and mortality. The association was more pronounced among patients aged ≥65 years across all three mortality endpoints, which may reflect the greater vulnerability of older AMI patients. Prior data have shown that reported contraindications to early beta-blocker use increase with age and are independently associated with higher in-hospital mortality.^23^ This pattern was consistent with the treatment-pattern comparator analysis, in which the association with mortality was strongest among patients receiving ivabradine without concomitant beta-blocker and was attenuated among those receiving ivabradine in addition to beta-blocker. These findings suggest that background beta-blocker use and treatment pattern should be considered when interpreting the association between ivabradine use and clinical outcomes. These subgroup and comparator findings should be interpreted as hypothesis-generating rather than confirmatory evidence of differential treatment effects.

The Kaplan–Meier curves show that the between-group prognostic difference emerges early after the index admission and is maintained largely unchanged throughout follow-up. This temporal pattern suggests that the observed 1-year mortality difference was concentrated in the early phase after AMI, with subsequent follow-up largely reflecting carry-over of this early prognostic separation rather than a continuously widening risk difference. This observation is consistent with the acute-phase exposure definition of the present study, in which ivabradine use was assessed during the index hospitalization for AMI. Because patients who initiated ivabradine only after discharge were excluded by design, the present analysis cannot determine whether later outpatient initiation of ivabradine carries a similar, weaker, neutral, or different risk profile. Two complementary explanations may account for the early separation of the survival curves. First, from a pharmacological perspective, during the haemodynamically unstable phase of AMI the myocardium is in a vulnerable state characterized by myocardial stunning, neurohormonal activation, abnormal loading conditions, and impaired coronary perfusion reserve; in this setting, ivabradine-induced heart rate reduction may interact unfavourably with acute haemodynamic vulnerability in selected patients, consistent with the central blood pressure mechanism discussed above and with previous haemodynamic data.^24^ Second, from an epidemiological perspective, post-AMI mortality follows a markedly time-dependent distribution, with risk being highest during the early period after the index event and declining thereafter.^25^ If an exposure-associated risk is concentrated predominantly within this acute window, it may produce an early separation in cumulative mortality that is subsequently carried forward during follow-up. The concordant increase in in-hospital mortality further supports the clinical relevance of this early safety signal. Nevertheless, given the observational design, residual confounding and confounding by indication cannot be excluded, and these findings should be interpreted as supporting cautious interpretation of in-hospital ivabradine use during the acute phase of myocardial infarction rather than as confirmatory evidence of direct harm.

The treatment-pattern comparator analysis further placed ivabradine use in the context of background beta-blocker therapy. Compared with patients receiving beta-blocker therapy without ivabradine, the association with mortality was strongest among patients receiving ivabradine without concomitant beta-blocker and was attenuated among those receiving ivabradine in addition to beta-blocker. These findings suggest that background beta-blocker use and treatment pattern should be considered when interpreting the association between ivabradine use and clinical outcomes. In parallel, achievement of conventional beta-blocker target doses was low in both treatment groups, and higher target-dose achievement was not clearly associated with lower mortality in our supplementary analyses. This finding is consistent with previous real-world AMI studies showing that beta-blockers are commonly prescribed after AMI but frequently used at subtarget doses. Arnold et al. reported that beta-blocker goal-dose achievement at 12 months was only 12%, while the OBTAIN registry showed that 91.5% of patients were discharged on beta-blockers but at a mean dose of only 38.1% of the target dose.^26,27^ Similarly, SWEDEHEART and KAMIR-NIH registry analyses found no clear additional prognostic benefit of higher vs. lower beta-blocker dose categories.^28,29^ Taken together, these data indicate that beta-blocker use, treatment pattern, and dose intensity in contemporary AMI care are complex and may not follow a simple dose-response relationship. Because the comparator and dose-intensity analyses were observational and exploratory, and because heart rate targets and physician-directed titration plans were not systematically captured, these findings should be interpreted descriptively and as hypothesis-generating rather than as evidence regarding optimal beta-blocker titration or causal treatment effects.

From a clinical perspective, these findings suggest that caution may be warranted when considering ivabradine initiation during hospitalization for AMI. Although the supplementary analyses showed clinically plausible associations for established AMI prognostic factors, the observational nature of the present study means that these findings should still be interpreted as a hypothesis-generating safety signal rather than as confirmatory evidence of harm. Future prospective studies and RCTs with systematic assessment of heart rate trajectories, beta-blocker titration, haemodynamic status, LVEF, functional capacity, and patient-reported quality-of-life outcomes are warranted to clarify the efficacy and safety profile of ivabradine in AMI and to identify patient subgroups in whom its use may be beneficial, neutral, or potentially harmful.

### Limitation

This study has several limitations. First, because this was an observational study, residual confounding and treatment-selection bias cannot be excluded despite PSM, multivariable adjustment, and weighted sensitivity analyses. In addition, subgroup and supplementary analyses were exploratory; therefore, the corresponding *P* values should be interpreted descriptively rather than confirmatorily. Second, several clinically important variables related to AMI severity and procedural management were not systematically available in the TCADSD, including detailed procedural-quality information for revascularization, residual SYNTAX score, and complete variables required to compute GRACE or TIMI risk scores. Therefore, we could not fully assess revascularization quality or calculate complete validated AMI prognostic scores. Third, several efficacy-related physiological and functional variables were also not systematically collected, including serial resting heart rate, post-discharge LVEF, NYHA functional class, 6-minute walk distance, exercise tolerance, and quality-of-life measures. Therefore, the present study could not comprehensively evaluate the physiological or functional efficacy of ivabradine after AMI. Future prospective studies with standardized assessment of heart rate trajectories, LVEF, beta-blocker titration, haemodynamic status, functional capacity, and quality-of-life outcomes are needed. Fourth, the present analysis specifically focused on ivabradine use during the index hospitalization for AMI. Patients who initiated ivabradine only after discharge were excluded to preserve the acute-phase exposure definition and reduce time-related bias. Therefore, our findings should not be extrapolated to patients who initiate ivabradine later during outpatient follow-up or the chronic post-AMI recovery phase.

## Conclusion

This multicentre, large-sample, real-world study indicates that in patients with AMI, the in-hospital use of ivabradine is associated with a higher risk of in-hospital mortality and 1-year cardiac and all-cause mortality. Given the observational design and the exploratory nature of several analyses, these findings should be interpreted as a hypothesis-generating safety signal rather than confirmatory evidence of harm. Prospective studies and RCTs are needed to confirm these findings and to clarify the patient populations in whom ivabradine may be beneficial, neutral, or potentially harmful after AMI.

## Supplementary Material

zuag084_Supplementary_Data

## Data Availability

The data that support the findings of this study are available from the corresponding authors upon reasonable request.
