# Supplementary material for: Safety and efficacy of ivabradine in the treatment of patients with acute myocardial infarction: a real-world propensity score matching study
Source: Eur Heart J Acute Cardiovasc Care. 2026 Jun 15;15(9):690–700. doi: 10.1093/ehjacc/zuag084 (PMC13624656; doi:10.1093/ehjacc/zuag084)
Supplement: Supplementary file 1 [file zuag084_supplementary_data.docx]

**Supplementary Material**

Safety and Efficacy of Ivabradine in Patients with Acute Myocardial Infarction: A Real-World Propensity Score Matching Study

**Supplementary Figure S1.** Standardized mean differences before and after propensity-score matching.

***Legend.*** Absolute standardized mean differences are shown for covariates included in the propensity-score model before and after matching. Grey circles represent SMDs before matching, and blue triangles represent SMDs after matching. The dashed vertical line indicates the prespecified balance threshold of SMD = 0.10. After propensity-score matching, all covariates achieved adequate balance between the ivabradine and control groups. ***Abbreviations:*** SMD = standardized mean difference.

**Supplementary Figure S2.** Subgroup analyses of the association between in-hospital ivabradine use and mortality outcomes in the propensity-score-matched cohort.

***Legend.*** Subgroup-stratified odds ratios or hazard ratios with 95% confidence intervals are shown for the association between in-hospital ivabradine use and mortality outcomes in the propensity-score-matched cohort. Odds ratios are presented for in-hospital mortality, and hazard ratios are presented for 1-year outcomes. Panel A shows in-hospital mortality; Panel B shows 1-year cardiac mortality; and Panel C shows 1-year all-cause mortality. ***Abbreviations:*** AMI = acute myocardial infarction; CI = confidence interval; NSTEMI = non-ST-elevation myocardial infarction; STEMI = ST-elevation myocardial infarction.

**Supplementary Figure S3.** Kaplan–Meier curves for 1-year mortality outcomes in the complete-case sensitivity analysis.

***Legend.*** Kaplan–Meier curves showing the cumulative incidence of 1-year cardiac mortality and 1-year all-cause mortality among patients with AMI treated with or without in-hospital ivabradine in the complete-case sensitivity analysis. The upper panel shows 1-year cardiac mortality, and the lower panel shows 1-year all-cause mortality. Red curves represent patients receiving in-hospital ivabradine, and blue curves represent patients not receiving ivabradine. Risk tables display the number at risk and the cumulative number of events at each time point. Between-group differences were assessed using the log-rank test. ***Abbreviations:*** AMI = acute myocardial infarction.

**Supplementary Table S1.** Doubly robust sensitivity analyses using inverse probability of treatment weighting and overlap weighting in the unmatched cohort.

| **Outcome** | **Effect measure** | **IPTW analysis** | | **Overlap weighting analysis** | |
| --- | --- | --- | --- | --- | --- |
|  |  | **Estimate (95% CI)** | **C-index** | **Estimate (95% CI)** | **C-index** |
| In-hospital mortality | aOR | 2.03 (1.48–2.79) | – | 1.60 (1.32–1.94) | – |
| 1-year all-cause mortality | aHR | 1.43 (1.22–1.67) | 0.849 | 1.25 (1.13–1.39) | 0.811 |
| 1-year cardiac mortality | aHR | 1.35 (1.12–1.61) | 0.858 | 1.22 (1.08–1.37) | 0.817 |

***Legend.*** Doubly robust outcome models combined propensity-score-derived weights with multivariable adjustment for prespecified confounders. Estimates are expressed for ivabradine use versus no ivabradine use. The IPTW analysis estimates the average treatment effect in the entire population (ATE), whereas the overlap weighting analysis estimates the average treatment effect in the overlap population (ATO). The concordance index (C-index) is reported only for the time-to-event Cox models. ***Abbreviations:*** aHR = adjusted hazard ratio; aOR = adjusted odds ratio; CI = confidence interval; C-index = concordance index; IPTW = inverse probability of treatment weighting.

**Supplementary Table S2.** E-value analyses for the primary propensity-score-matched (PSM) Model 4 estimates.

| **Outcome** | **E-value (point estimate)** | **E-value (lower 95% CI bound)** |
| --- | --- | --- |
| In-hospital mortality | 2.32 | 1.46 |
| 1-year all-cause mortality | 1.74 | 1.28 |
| 1-year cardiac mortality | 1.71 | 1.11 |

***Legend.*** E-values represent the minimum strength of association, on the risk-ratio scale, that an unmeasured confounder would need to have with both in-hospital ivabradine use and the outcome, conditional on measured covariates, to fully explain away the observed ivabradine–outcome association. ***Abbreviations:*** CI = confidence interval; PSM = propensity-score matching.

**Supplementary Table S3.** Treatment-pattern comparator analysis using patients receiving beta-blocker alone as the reference.

| **Group** | **N** | **In-hospital mortality, n (%)** | **1-year cardiac mortality, n (%)** | **1-year all-cause mortality, n (%)** | **In-hospital aOR (95% CI)** | **1-year cardiac mortality aHR (95% CI)** | **1-year all-cause mortality aHR (95% CI)** |
| --- | --- | --- | --- | --- | --- | --- | --- |
| Beta-blocker without ivabradine (reference) | 90,830 | 1,539 (1.7) | 4,460 (4.9) | 5,949 (6.5) | Reference | Reference | Reference |
| Ivabradine without beta-blocker | 602 | 69 (11.5) | 115 (19.1) | 161 (26.7) | 3.20 (2.38–4.24), P < 0.001 | 1.87 (1.55–2.25), P < 0.001 | 2.07 (1.77–2.42), P < 0.001 |
| Ivabradine + beta-blocker | 2,510 | 81 (3.2) | 200 (8.0) | 256 (10.2) | 1.50 (1.17–1.90), P = 0.001 | 1.21 (1.05–1.39), P = 0.010 | 1.18 (1.04–1.34), P = 0.011 |

***Legend.*** Patients receiving a beta-blocker without ivabradine served as the reference group. Two treatment patterns involving ivabradine were compared: ivabradine without concomitant beta-blocker and ivabradine in addition to beta-blocker. Estimates are expressed relative to beta-blocker use without ivabradine. Multivariable adjustment included sex, age, STEMI status, Killip class, the Charlson Comorbidity Index, the Hospital Frailty Risk Score, comorbidities, and other in-hospital medications. ***Abbreviations:*** aHR = adjusted hazard ratio; aOR = adjusted odds ratio; CI = confidence interval.

**Supplementary Table S4.** Univariable and multivariable logistic regression analyses for in-hospital mortality in the overall study cohort.

| **Variable** | **Univariable analysis** | | **Multivariable analysis** | |
| --- | --- | --- | --- | --- |
|  | **OR (95% CI)** | ***P* value** | **OR (95% CI)** | ***P* value** |
| Male sex | 0.64 (0.60–0.68) | < 0.001 | 1.17 (1.07–1.27) | < 0.001 |
| Age (per 1-year increase) | 1.07 (1.07–1.07) | < 0.001 | 1.03 (1.03–1.04) | < 0.001 |
| STEMI | 1.14 (1.08–1.21) | < 0.001 | 1.71 (1.56–1.87) | < 0.001 |
| *Killip class (reference: class I)* |  |  |  |  |
| Killip class II | 1.97 (1.80–2.16) | < 0.001 | 1.57 (1.38–1.79) | < 0.001 |
| Killip class III | 7.07 (6.53–7.66) | < 0.001 | 3.20 (2.84–3.62) | < 0.001 |
| Killip class IV | 27.87 (25.72–30.21) | < 0.001 | 10.49 (9.18–11.98) | < 0.001 |
| Charlson Comorbidity Index (per 1-point) | 1.21 (1.19–1.22) | < 0.001 | 1.08 (1.06–1.10) | < 0.001 |
| Hospital Frailty Risk Score (per 1-point) | 1.21 (1.20–1.22) | < 0.001 | 1.04 (1.03–1.05) | < 0.001 |
| Hypertension | 1.08 (1.02–1.15) | 0.011 | 1.01 (0.93–1.10) | 0.812 |
| Diabetes mellitus | 1.30 (1.22–1.37) | < 0.001 | 1.11 (1.01–1.21) | 0.028 |
| Cerebrovascular disease | 2.09 (1.97–2.22) | < 0.001 | 1.05 (0.95–1.16) | 0.309 |
| In-hospital PCI | 0.12 (0.11–0.13) | < 0.001 | 0.38 (0.33–0.42) | < 0.001 |
| In-hospital β-blocker use | 0.21 (0.20–0.23) | < 0.001 | 0.61 (0.56–0.67) | < 0.001 |
| In-hospital ACEI/ARB use | 0.15 (0.14–0.16) | < 0.001 | 0.39 (0.36–0.43) | < 0.001 |
| In-hospital CCB use | 0.74 (0.68–0.80) | < 0.001 | 0.91 (0.82–1.02) | 0.119 |
| In-hospital statin use | 0.11 (0.11–0.12) | < 0.001 | 0.43 (0.39–0.47) | < 0.001 |
| In-hospital ivabradine use | 1.38 (1.16–1.62) | < 0.001 | 1.75 (1.41–2.15) | < 0.001 |

***Legend.*** Univariable models included each variable individually, and the multivariable model included all listed variables simultaneously. For continuous variables, estimates correspond to a one-unit increase. For Killip class, class I served as the reference category. For binary variables, the reference categories were female sex for male sex, NSTEMI for STEMI, and absence of the listed comorbidity or in-hospital treatment for the remaining variables. Analyses were performed in the overall pre-matching cohort. ***Abbreviations:*** ACEI = angiotensin-converting enzyme inhibitor; ARB = angiotensin receptor blocker; CCB = calcium channel blocker; CI = confidence interval; NSTEMI = non-ST-elevation myocardial infarction; OR = odds ratio; PCI = percutaneous coronary intervention; STEMI = ST-elevation myocardial infarction.

**Supplementary Table S5.** Univariable and multivariable Cox proportional hazards regression analyses for 1-year cardiac mortality in the overall study cohort.

| **Variable** | **Univariable analysis** | | **Multivariable analysis** | |
| --- | --- | --- | --- | --- |
|  | **HR (95% CI)** | ***P* value** | **HR (95% CI)** | ***P* value** |
| Male sex | 0.56 (0.54–0.59) | < 0.001 | 1.06 (1.01–1.12) | 0.017 |
| Age (per 1-year increase) | 1.08 (1.07–1.08) | < 0.001 | 1.03 (1.03–1.03) | < 0.001 |
| STEMI | 1.02 (0.98–1.06) | 0.290 | 1.28 (1.21–1.35) | < 0.001 |
| *Killip class (reference: class I)* |  |  |  |  |
| Killip class II | 2.42 (2.29–2.56) | < 0.001 | 1.48 (1.38–1.60) | < 0.001 |
| Killip class III | 6.30 (5.98–6.63) | < 0.001 | 2.40 (2.23–2.59) | < 0.001 |
| Killip class IV | 18.06 (17.10–19.06) | < 0.001 | 5.48 (5.05–5.95) | < 0.001 |
| Charlson Comorbidity Index (per 1-point) | 1.21 (1.20–1.21) | < 0.001 | 1.03 (1.02–1.05) | < 0.001 |
| Hospital Frailty Risk Score (per 1-point) | 1.19 (1.19–1.20) | < 0.001 | 1.03 (1.02–1.03) | < 0.001 |
| Hypertension | 1.12 (1.08–1.17) | < 0.001 | 1.06 (1.00–1.12) | 0.041 |
| Diabetes mellitus | 1.39 (1.33–1.44) | < 0.001 | 1.13 (1.07–1.20) | < 0.001 |
| Cerebrovascular disease | 2.21 (2.13–2.30) | < 0.001 | 1.14 (1.08–1.21) | < 0.001 |
| In-hospital PCI | 0.12 (0.11–0.13) | < 0.001 | 0.31 (0.29–0.34) | < 0.001 |
| In-hospital β-blocker use | 0.36 (0.34–0.37) | < 0.001 | 0.78 (0.74–0.82) | < 0.001 |
| In-hospital ACEI/ARB use | 0.29 (0.28–0.30) | < 0.001 | 0.59 (0.56–0.62) | < 0.001 |
| In-hospital CCB use | 0.95 (0.91–1.00) | 0.052 | 0.82 (0.77–0.87) | < 0.001 |
| In-hospital statin use | 0.20 (0.19–0.21) | < 0.001 | 0.61 (0.58–0.65) | < 0.001 |
| In-hospital ivabradine use | 1.36 (1.22–1.53) | < 0.001 | 1.24 (1.09–1.42) | 0.001 |

***Abbreviations:*** ACEI = angiotensin-converting enzyme inhibitor; ARB = angiotensin receptor blocker; CCB = calcium channel blocker; CI = confidence interval; NSTEMI = non-ST-elevation myocardial infarction; OR = odds ratio; PCI = percutaneous coronary intervention; STEMI = ST-elevation myocardial infarction.

**Supplementary Table S6.** Univariable and multivariable Cox proportional hazards regression analyses for 1-year all-cause mortality in the overall study cohort.

| **Variable** | **Univariable analysis** | | **Multivariable analysis** | |
| --- | --- | --- | --- | --- |
|  | **HR (95% CI)** | ***P* value** | **HR (95% CI)** | ***P* value** |
| Male sex | 0.58 (0.56–0.60) | < 0.001 | 1.06 (1.01–1.11) | 0.017 |
| Age (per 1-year increase) | 1.08 (1.07–1.08) | < 0.001 | 1.03 (1.03–1.03) | < 0.001 |
| STEMI | 1.06 (1.02–1.09) | 0.002 | 1.30 (1.24–1.36) | < 0.001 |
| *Killip class (reference: class I)* |  |  |  |  |
| Killip class II | 2.33 (2.22–2.44) | < 0.001 | 1.40 (1.32–1.50) | < 0.001 |
| Killip class III | 5.79 (5.53–6.05) | < 0.001 | 2.16 (2.02–2.30) | < 0.001 |
| Killip class IV | 15.04 (14.32–15.78) | < 0.001 | 4.56 (4.24–4.91) | < 0.001 |
| Charlson Comorbidity Index (per 1-point) | 1.23 (1.22–1.23) | < 0.001 | 1.06 (1.05–1.07) | < 0.001 |
| Hospital Frailty Risk Score (per 1-point) | 1.20 (1.19–1.20) | < 0.001 | 1.03 (1.03–1.04) | < 0.001 |
| Hypertension | 1.11 (1.07–1.15) | < 0.001 | 1.02 (0.98–1.08) | 0.335 |
| Diabetes mellitus | 1.39 (1.34–1.44) | < 0.001 | 1.09 (1.04–1.14) | < 0.001 |
| Cerebrovascular disease | 2.26 (2.19–2.34) | < 0.001 | 1.12 (1.07–1.18) | < 0.001 |
| In-hospital PCI | 0.12 (0.11–0.12) | < 0.001 | 0.31 (0.29–0.33) | < 0.001 |
| In-hospital β-blocker use | 0.37 (0.36–0.38) | < 0.001 | 0.77 (0.73–0.81) | < 0.001 |
| In-hospital ACEI/ARB use | 0.31 (0.30–0.32) | < 0.001 | 0.62 (0.59–0.65) | < 0.001 |
| In-hospital CCB use | 1.07 (1.03–1.12) | < 0.001 | 0.89 (0.84–0.94) | < 0.001 |
| In-hospital statin use | 0.20 (0.19–0.21) | < 0.001 | 0.61 (0.58–0.65) | < 0.001 |
| In-hospital ivabradine use | 1.39 (1.26–1.53) | < 0.001 | 1.24 (1.10–1.39) | < 0.001 |

***Abbreviations:*** ACEI = angiotensin-converting enzyme inhibitor; ARB = angiotensin receptor blocker; CCB = calcium channel blocker; CI = confidence interval; NSTEMI = non-ST-elevation myocardial infarction; OR = odds ratio; PCI = percutaneous coronary intervention; STEMI = ST-elevation myocardial infarction.

**Supplementary Table S7.** Beta-blocker target-dose achievement among patients with available dose data according to ivabradine status.

| ***Dose achievement by ivabradine status*** | **Number of beta-blocker user** | **≥50% target dose, n (%)** | **≥100% target dose, n (%)** |
| --- | --- | --- | --- |
| No ivabradine | 90,649 | 27,274 (30.1) | 2,850 (3.1) |
| Ivabradine | 2,503 | 826 (33.0) | 99 (4.0) |

***Legend.*** Among patients with available beta-blocker dose data, the proportions achieving ≥50% and ≥100% of the guideline- or trial-based target dose were compared between ivabradine and non-ivabradine users.

**Supplementary Table S8.** Association of beta-blocker target-dose achievement with in-hospital and 1-year outcomes.

| **Beta-blocker dose category** | **N** | **In-hospital mortality, n (%)** | **1-yr cardiac** **mortality, n (%)** | **1-yr all-cause** **mortality, n (%)** | **In-hospital** **mortality aOR (95% CI)** | **1-yr cardiac mortality aHR (95% CI)** | **1-yr all-cause mortality aHR (95% CI)** |
| --- | --- | --- | --- | --- | --- | --- | --- |
| <50% (reference) | 65,052 | 1,150 (1.8) | 3,262 (5.0) | 4,308 (6.6) | Reference | Reference | Reference |
| ≥50% | 28,100 | 460 (1.6) | 1,378 (4.9) | 1,867 (6.6) | 0.90 (0.80–1.01), P = 0.084 | 0.96 (0.90–1.02),  P = 0.188 | 0.98 (0.93–1.03),  P = 0.432 |
| <100% (reference) | 90,218 | 1547 (1.7%) | 4486 (5.0%) | 5961 (6.6%) | Reference | Reference | Reference |
| ≥100% | 2,934 | 63 (2.1%) | 154 (5.2%) | 214 (7.3%) | 1.09 (0.82-1.41), P=0.547 | 0.92 (0.79-1.08),  P=0.334 | 0.96 (0.84-1.10),  P=0.576 |

***Legend.*** The association between beta-blocker dose intensity and clinical outcomes was assessed among beta-blocker users with available dose data using two thresholds: ≥50% versus <50% of target dose and ≥100% versus <100% of target dose. Multivariable models adjusted for sex, age, STEMI status, Killip class, the Charlson Comorbidity Index, the Hospital Frailty Risk Score, comorbidities, and other in-hospital medications. ***Abbreviations:*** aHR = adjusted hazard ratio; aOR = adjusted odds ratio; CI = confidence interval.

**Supplementary Table S9.** Complete-case sensitivity analysis of in-hospital and 1-year adverse outcomes.

| **Outcome** | **Unmatched cohort** | | **PS-matched cohort** | |
| --- | --- | --- | --- | --- |
|  | **Event rate (%)** | **aOR/aHR (95% CI), P** | **Event rate (%)** | **aOR/aHR (95% CI), P** |
| ***In-hospital adverse events*** |  |  |  |  |
| In-hospital mortality | 5.1 vs 3.6 | 1.75 (1.41–2.16), P < 0.001 | 5.2 vs 3.9 | 1.36 (1.03–1.81), P = 0.033 |
| Rapid malignant arrhythmias | 5.3 vs 3.3 | 1.22 (1.00–1.48), P = 0.050 | 5.2 vs 4.8 | 1.10 (0.84–1.44), P = 0.496 |
| ***1-year adverse events*** |  |  |  |  |
| Cardiac mortality | 10.2 vs 7.8 | 1.24 (1.09–1.42), P = 0.001 | 10.3 vs 7.5 | 1.39 (1.14–1.69), P < 0.001 |
| All-cause mortality | 13.5 vs 10.2 | 1.24 (1.10–1.39), P < 0.001 | 13.6 vs 10.3 | 1.33 (1.12–1.57), P < 0.001 |
| Myocardial infarction | 5.5 vs 4.0 | 1.02 (0.85–1.22), P = 0.831 | 5.5 vs 4.3 | 1.04 (0.72–1.77), P = 0.476 |
| Stroke | 3.0 vs 2.3 | 0.98 (0.60–1.45), P = 0.409 | 3.0 vs 2.1 | 1.01 (0.86–2.06), P = 0.641 |
| Revascularization | 16.6 vs 15.9 | 1.09 (0.98–1.21), P = 0.097 | 16.6 vs 15.9 | 1.05 (0.91–1.22), P = 0.480 |
| BARC ≥ 3 bleeding | 1.2 vs 0.8 | 1.24 (0.84–1.81), P = 0.278 | 1.2 vs 1.1 | 1.14 (0.66–1.95), P = 0.646 |

***Legend.*** Event rates are shown as ivabradine versus no ivabradine. Estimates are expressed for ivabradine use versus no ivabradine use. Logistic regression was used for in-hospital outcomes, and Cox proportional hazards models were used for 1-year outcomes. ***Abbreviations***: aHR = adjusted hazard ratio; aOR = adjusted odds ratio; BARC = Bleeding Academic Research Consortium; CI = confidence interval; PS = propensity score.

**Supplementary Table S10.** Complete-case sensitivity analysis using nested multivariable models for primary outcomes in the propensity-score-matched cohort.

| **Model** | **In-hospital mortality** | | **1-year all-cause mortality** | | **1-year cardiac mortality** | |
| --- | --- | --- | --- | --- | --- | --- |
|  | **aOR (95% CI)** | ***P* value** | **aHR (95% CI)** | ***P* value** | **aHR (95% CI)** | ***P* value** |
| Model 1 | 1.37 (1.01–1.85) | 0.043 | 1.28 (1.07–1.53) | 0.006 | 1.33 (1.08–1.63) | 0.006 |
| Model 2 | 1.39 (1.03–1.89) | 0.034 | 1.29 (1.08–1.54) | 0.005 | 1.33 (1.09–1.64) | 0.006 |
| Model 3 | 1.48 (1.08–2.04) | 0.016 | 1.28 (1.07–1.53) | 0.006 | 1.34 (1.09–1.65) | 0.005 |
| Model 4 | 1.54 (1.12–2.13) | 0.009 | 1.29 (1.08–1.54) | 0.005 | 1.35 (1.10–1.66) | 0.004 |

***Legend.*** Estimates are expressed for ivabradine use versus no ivabradine use. Model 1 included sex, age, STEMI status, Killip class, the Charlson Comorbidity Index, and the Hospital Frailty Risk Score; Model 2 additionally adjusted for comorbidities at admission; Model 3 additionally adjusted for in-hospital pharmacotherapy; and Model 4 additionally adjusted for the location of the index myocardial infarction and additional baseline biomarkers, including creatine kinase-MB, estimated glomerular filtration rate, and haemoglobin. ***Abbreviations:*** aHR = adjusted hazard ratio; aOR = adjusted odds ratio; CI = confidence interval.
